# Effect of Linagliptin and Voglibose on metabolic profile in patients with Type 2 Diabetes: a randomized, double-blind, placebo-controlled trial

**DOI:** 10.1186/s40360-018-0228-z

**Published:** 2018-07-03

**Authors:** Girish Parthan, Shobhit Bhansali, Anura V. Kurpad, Rama Walia, Kishor Bhat, Anil Bhansali

**Affiliations:** 1 Department of Endocrinology, Post Graduate Institute of Medical Education and Research, Sector- 12, Chandigarh, PIN Code-160012 India; 2 Department of Physiology, St. John’s Medical College, Bangalore, India

**Keywords:** T2DM, β-cell function, insulin sensitivity, linagliptin, voglibose

## Abstract

**Background:**

Dipeptidyl peptidase 4 (DPP4) inhibitors improve glycemic control by promoting GLP1-mediated glucose-dependent insulin secretion and suppression of glucagon. Sitagliptin and vildagliptin have been shown to improve insulin sensitivity in patients with type 2 diabetes mellitus (T2DM). However, these patients had uncontrolled blood glucose at inclusion; therefore, the improvement in insulin sensitivity observed in these studies could be attributed to the drug per se and/or reduction in glucotoxicity. This study examines the effect of linagliptin on insulin sensitivity and β-cell function in patients with well-controlled T2DM.

**Methods:**

Thirty patients with T2DM of duration ≤5 years, and having HbA1c < 7.5% were randomized to receive linagliptin, voglibose or placebo (*n* = 10 each), and were followed up for 6 months. Insulin sensitivity was assessed by hyperinsulinemic-euglycemic clamp, and insulin secretory response was measured by basal (M_0_) and postprandial (M_1_) β-cell function, and area under curve (AUC) for C-peptide during mixed meal tolerance test.

**Results:**

The median HbA1c of the study subjects at inclusion was 6.9% and there was no significant difference among the groups in terms of age, duration of diabetes, body mass index (BMI), HbA1c, insulin sensitivity, AUC of C-peptide and M_0_ and M_1_ at baseline. At the end of the study, there was a modest reduction in HbA1c (− 0.2%) in the linagliptin group, and a significant decrease (− 0.8%) in the voglibose group, as compared to placebo (*p* = 0.038). However, there were no significant differences in insulin sensitivity, M_0_ and M_1_ and AUC of C-peptide, within, or among the groups.

**Conclusion:**

Linagliptin modestly improves glycemic profile in patients with well controlled T2DM; however, it may not have an effect on insulin sensitivity in these patients.

**Trial registration:**

Retrospectively Registered in Clinicaltrials.gov (ID number, NCT02097342). Registered: March 27, 2014.

## Background

The prevalence of diabetes is increasing worldwide and is fast expanding from developed countries to developing countries. As per an estimate by the International Diabetes Federation, 414 million adults were affected with diabetes in 2015, and this number is likely to swell to 642 million by 2040 [1].

Type 2 diabetes mellitus (T2DM) is characterized by two cardinal defects; insulin resistance and insulin deficiency. A plethora of drugs are available for the treatment of T2DM; incretin-based therapies such as dipeptidyl peptidase-4(DPP-4) inhibitors and glucagon like peptide-1(GLP-1) receptor agonists are among the recent additions to the therapeutic armamentarium in T2DM [2].

DPP-4 inhibitors are orally acting drugs that have efficacy similar to sulfonylureas, but without the risk of hypoglycemia or weight gain. DPP-4 inhibitors are commonly used as second-line drugs in the management of T2DM, although they have been found to be useful as monotherapy, as well [3]. These drugs enhance the effect of endogenous GLP-1 by preventing its degradation, and this results in augmentation of glucose-dependent insulin secretion and suppression of glucagon, thereby reducing blood glucose. DPP-4 inhibitors are also known to improve pancreatic β-cell function [4–7]. In addition, DPP-4 has also been shown to be an adipokine, which impairs insulin sensitivity in an autocrine and paracrine fashion [8]; hence, DPP-4 inhibition may improve insulin sensitivity. Previously, few studies have reported an improvement in insulin sensitivity with DPP-4 inhibitors, sitagliptin and vildagliptin [9, 10]. However, these studies included patients with uncontrolled diabetes (HbA1c > 8%) and one of the potential reasons for the improvement in insulin sensitivity observed in these studies could be due to reduction in glucotoxicity, rather than the direct effect of these drugs on peripheral utilization of glucose. Recently, linagliptin has been shown to improve insulin sensitivity in diet-induced obese mice [11].

There are various methods to assess the insulin sensitivity like insulin tolerance test, frequently sampled intravenous glucose tolerance test, quantitative insulin sensitivity check index (QUICKI), mixed meal tolerance test, Matsuda index and homeostasis model assessment-estimated insulin resistance (HOMA–IR) [12]. However, the “gold standard” for the assessment of insulin resistance is hyperinsulinemic euglycemic clamp study [13]. Till date, no study has evaluated the effect of linagliptin on insulin sensitivity in humans by using hyperinsulinemic-euglycemic clamp study. This is a pilot study which aimed to evaluate the effect of linagliptin on insulin sensitivity and β-cell function by using hyperinsulinemic euglycemic clamp and mixed meal tolerance test, respectively, in patients with well controlled T2DM.

## Methods

### Study design

Patients with T2DM, aged between 30 and 65 years, with duration of diabetes < 5 years, and were on metformin 2 g/day for at least 6 weeks, with HbA1c < 7.5% (< 58.0 mmol/mol) were included in the study. Patients with history of ketoacidosis, abnormal liver function tests (plasma aminotransferase elevations of more than 3 times upper limit of normal), renal failure (serum creatinine more than 1.5 mg/dL), macular edema, coronary artery disease or heart failure, cerebrovascular disease, and those requiring insulin were excluded. Pregnant and lactating women, and those who received any DPP-4 inhibitors in the last 3 months were also excluded. All patients were educated about their disease and were advised life-style modifications. Informed consent was obtained from the study subjects. Institute Ethics Committee approved the study, and the trial was registered at Clinicaltrials.gov (ID number, NCT02097342). Patients were allocated into three groups by random allocation software and received linagliptin 5 mg once daily (OD), placebo OD or voglibose (0.2 mg three times daily), and were followed up for 6 months. Voglibose was used as an active comparator to evaluate the effects of linagliptin on insulin sensitivity independent of alterations in HbA1c. Both patients and physicians were blinded to treatment, but the active comparator group of voglibose was open-labeled, as it was given three times a day.

### Baseline evaluation

Baseline evaluation included clinical examination, and biochemical assessment of glycemic control and evaluation for micro- and macrovascular complications.

Fasting plasma glucose (FPG), C-peptide, homeostasis model assessment estimated insulin resistance index (HOMA-IR), homeostasis model assessment β-cell function index (HOMA-β) and hyperinsulinemic euglycemic clamp study were performed at baseline. A mixed meal tolerance study was also done at baseline, after a week of HEC. Laboratory investigations were performed between 8 and 9 am after an overnight fast, and venous blood samples were collected in ethylenediaminetetraacetic acid (EDTA) vacutainer. Glycated hemoglobin (HbA1c) was estimated by an automated high-performance liquid chromatography (HPLC)-based system using ion-exchange cartridge (D-10, Bio-Rad Laboratories, Inc., Hercules, CA, USA). C-peptide estimation was done by electrochemiluminiscence immunoassay (ECLIA) (Elecsys 2010, Roche Diagnostics GmbH, Mannheim, Germany).

### Estimation of homeostatic model assessment

The new version of the homeostatic model assessment of insulin resistance (HOMA-IR) and β cell function (HOMA-β) was estimated as per the standard formulae [14, 15].

### Hyperinsulinemic-Euglycemic clamp study

Hyperinsulinemic-euglycemic clamp study was performed in all subjects to assess in-vivo insulin sensitivity. Subjects were requested to refrain from vigorous exercise, and reported at 0630 h after an overnight fast of 10 h. An intravenous catheter was inserted into the antecubital vein for infusion of insulin and 25% dextrose solutions, while another catheter was inserted in an anti-flow direction into the dorsal vein of the contralateral hand for arterialized blood sampling. An insulin infusate of 300 mU/ml was prepared from regular human insulin (Eli Lilly & Co. Gurugram, India) in 100 ml isotonic saline and 4 ml of subject’s blood. Insulin was infused intravenously based on the surface area at a constant rate (40 mU/m^2^/min) to raise the plasma insulin concentration to about 100 μU/mL. The glucose infusion rate was adjusted to maintain a steady state of 4.9 mmol/L. Blood samples for glucose and insulin were collected every 5 and 20 min, respectively. Plasma glucose was analysed by glucose oxidase method on a bedside glucose analyzer (GM9D, Analox instruments, London, UK). M value, a measure of glucose utilization, and M/I, an index of insulin sensitivity were calculated over 40 to 120 min of the clamp study [13].

### Mixed meal tolerance test

The mixed meal at a dose of 10 Kcal/Kg, (Ensure, Abbott Nutrition, Abbott Laboratories, India) was dissolved in 500 mL of water and was consumed within 10 min. Medications taken by the subject in the morning were administered 20 min prior to the start of the test meal. C-peptide concentrations were estimated at 0, 30, 60, 90, 120, 150 and 180 min. Total area under curve (AUC) for C-peptide from pre-meal to 180 min was calculated.

### Insulin secretion model

Basal β-cell function (BBCF) and postprandial β-cell function (PBCF) were assessed from glucose and C-peptide time-concentration profiles during the MMTT using an insulin secretion model [16]. M_0_ is an index of the BBCF and represents the ability of fasting plasma glucose to stimulate the β-cell. M_I_ is an index of PBCF and represents the ability of postprandial glucose to step up β-cell secretion. It equals the increment in insulin secretion in response to a unit increment in plasma glucose concentration.

### Follow-up

All patients were followed up for a period of 6 months. Lifestyle modification advice was reinforced during each visit to all the patients. All concomitant medications were continued throughout the study period without any dose modifications. Biochemical parameters and hyperinsulinemic-euglycemic clamp study and mixed meal tolerance test were repeated after 6 months.

### Statistical analysis

All data are expressed as median and interquartile range. Baseline and post-treatment data within the groups were compared using Wilcoxon’s signed rank test. The data between the groups were analyzed using the Kruskal Wallis test followed by Mann-Whitney test for two groups (*P* value corrected using Bonferroni procedure). A *p* value < 0.05 was considered significant. The statistical analysis was carried out using the SPSS version 22 for Windows (SPSS Inc., Chicago, USA).

## Results

### Study subjects

**Fig. 1:**
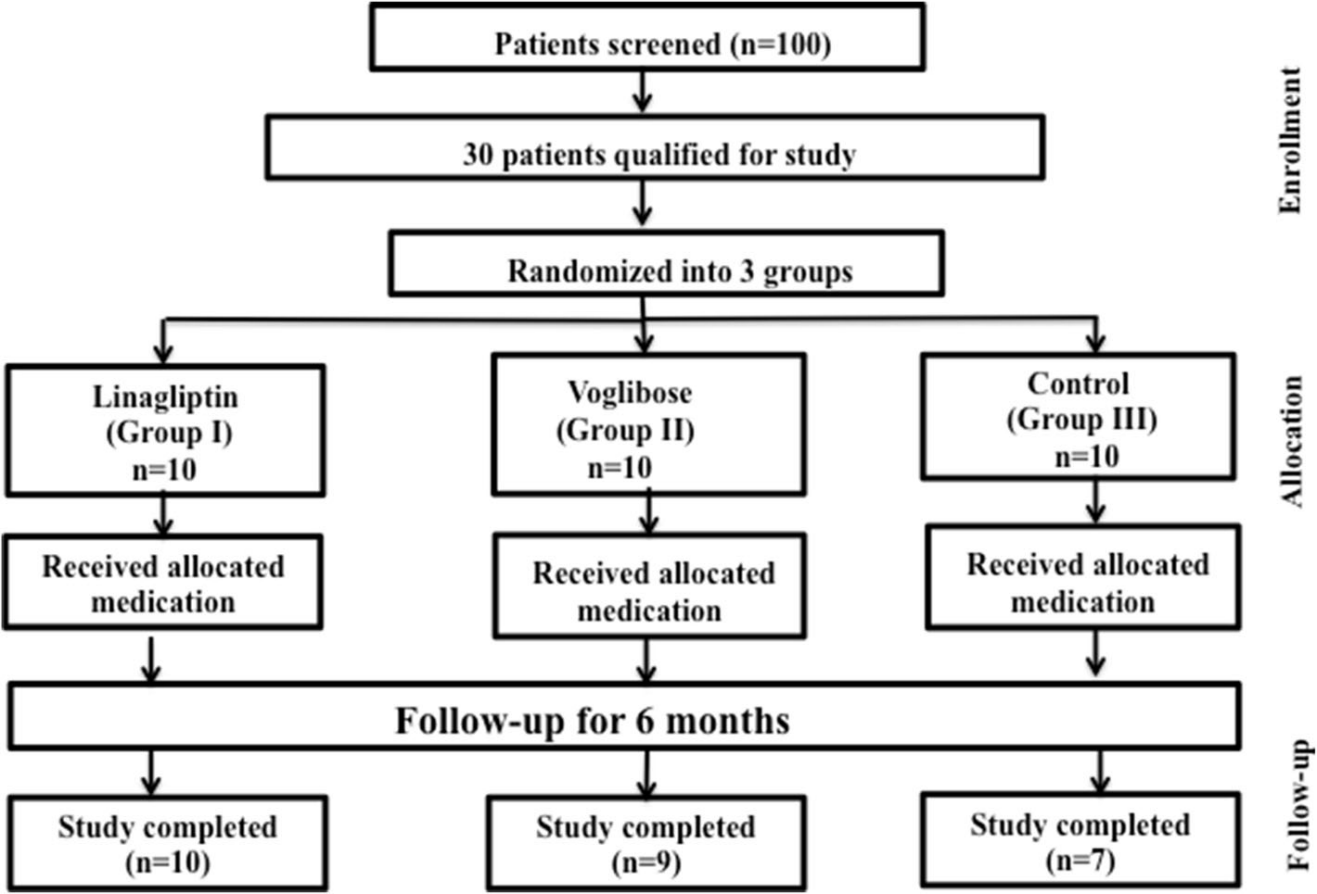
Schema of the study

Twenty-six out of thirty patients completed the study; 10 in the linagliptin group, 9 in the voglibose group and 7 in the placebo group (Fig. 1). One patient in placebo and voglibose group was excluded due to gastrointestinal side-effects, while two patients withdrew their consent for study in the placebo group. The baseline clinical and biochemical characteristics of the three groups were similar (Table 1).

**Table 1:** Baseline clinical and biochemical parameters of the study groups

Parameters	Linagliptin Group (*n* = 10)	Voglibose Group (*n* = 9)	Control Group (*n* = 7)	*P*-value
Age (years)	49.5 (41.5–55.5)	50.0 (45.0–54.0)	54.0 (48.5–59.5)	0.438
Sex (M:F)	5:5	7:2	5:2	
Duration of diabetes (years)	2.5 (1.0–3.0)	2.5 (0.8 to 3.0)	2.0 (0.8–2.8)	0.813
Weight (Kg)	75.3 (69.2–85.3)	60.7 (55.4–71.5)	74.0 (58.7–84.4)	0.071
BMI (Kg/m^2^)	25.5 (23.6–31.1)	25.9 (23.8–26.6)	26.4 (23.8–30.5)	0.834
HbA1c (%)	6.8 (6.4–7.1)	7.0 (6.8–7.4)	7.0 (6.8–7.1)	0.350
Fasting Plasma Glucose (mmol/L)	6.5 (5.9–7.3)	6.9 (6.2–7.4)	6.2 (6.1–6.6)	0.287
AUC C-peptide (nmol/L)	473.3 (376.9–567.8)	421.2 (40.9.8–526.9)	595.6 (460.5–623.8)	0.223
M_0_ × 10^− 8^ (1/min)	−0.4(− 2.6–0.8)	− 0.5 (− 2.8–1.2)	−2.6(− 5.9–1.2)	0.834
M_1_ ×10^−8^ (1/min)	6.4 (4.5–8.7)	5.7 (3.6–7.6)	7.7 (5.5–10.6)	0.319
Glucose disposal rate (mg/Kg min)	2.7 (2.0–5.5)	3.2 (2.6–3.5)	2.2 (1.8–3.0)	0.297
Insulin sensitivity (mg/(Kgmin)/μU/mL)	4.4 (2.5–8.0)	5.3 (3.6–6.7)	3.2 (2.3–5.1)	0.315
HOMA-IR	2.0 (1.9–2.3)	1.6 (1.3–2.0)	1.9 (1.6–2.1)	0.261
HOMA-β (%)	84.1 (75.1–95.9)	65.0 (52.2–85.7)	92.5 (82.6–102.1)	0.067

All values are expressed as median and interquartile range (1st IQR – 3rd IQR)

*AUC* Area under curve, *M*_*0*_ Basal β-cell function, *M*_*1*_ postprandial β-cell function, *HOMA-IR* Homeostatic model assessment of insulin resistance, and *HOMA-β* Homeostatic model assessment of β cell function

### Efficacy

**Fig. 2:**
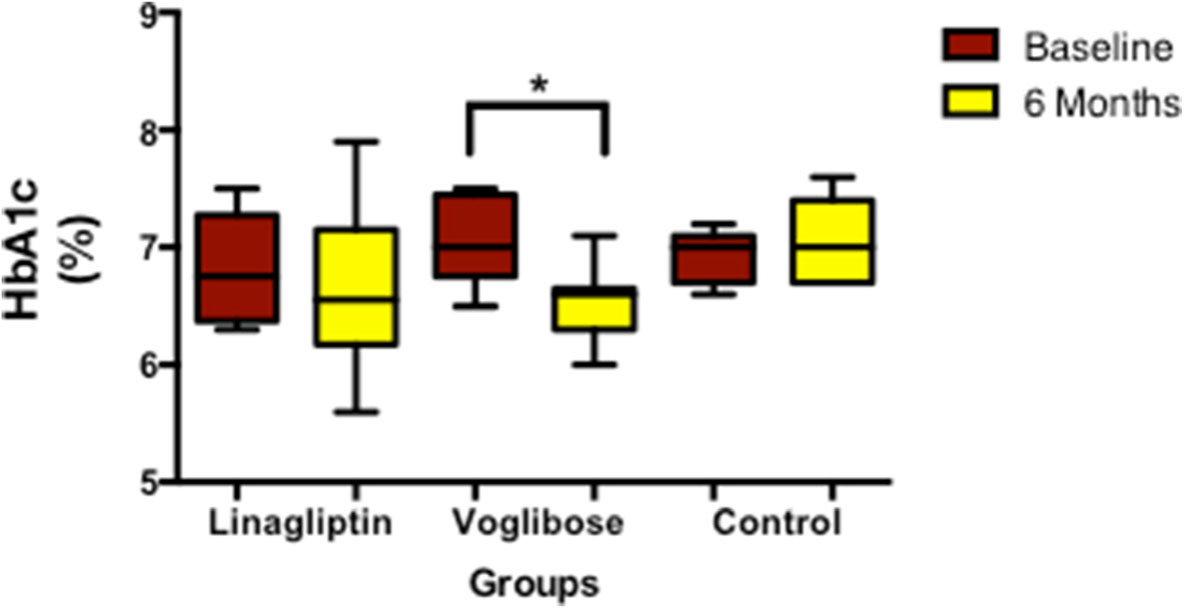
Change in HbA1c in the study groups. *Means significant difference from baseline

The median HbA1c of the study subjects at baseline was 6.9% and at the end of study it reduced to 6.6%. After 6 months of linagliptin and voglibose therapy reduced the HbA1c from 6.8 to 6.6% (*p* = 0.474) and 7.0 to 6.6%; *p* = 0.015), respectively, whereas in the control group, HbA1c did not change (Table 2, Fig. 2).

**Table 2:** Clinical and biochemical parameters within the groups

	Linagliptin Group			Voglibose Group			Control Group		
Parameters	Baseline (*n* = 10)	6 months (*n* = 10)	*P* value	Baseline (*n* = 9)	6 months (*n* = 9)	*P* value	Baseline (*n* = 7)	6 months (*n* = 7)	*P* value
Weight (Kg)	75.3 (69.2–85.3)	73.4(66.3–84.3)	0.066	60.7(55.4–71.5)	56.8(53.5–67.7)	0.208	74.0(58.7–84.4)	75.3(57.9–82.4)	0.612
BMI (kg/m^2^)	25.5 (23.6–31.1)	25.0(23.1–30.1)	0.093	25.9(23.8–26.6)	24.4(23.8–25.7)	0.260	26.4(23.8–30.5)	26.6(23.7–30.7)	0.612
HbA1c (%)	6.8 (6.4–7.1)	6.6 (6.3–6.9)	0.474	7.0(6.8–7.4)	6.6 (6.5–6.6)	0.015^a^	7.0(6.8–7.1)	7.0(6.8–7.4)	0.491
Fasting Plasma Glucose (mmol/L)	6.5(5.9–7.3)	6.3(5.6–6.9)	0.308	6.9(6.2–7.4)	6.3(5.8–6.6)	0.058	6.2(6.1–6.6)	6.9(6.2–7.4)	0.310
HOMA-IR	2.0(1.9–2.3)	1.7(1.1–2.1)	0.169	1.6(1.3–2.0)	1.7(1.2–1.9)	0.678	2.4(1.7–3.3)	0.128	0.128
HOMA-β (%)	84.1(75.1–95.9)	81.9(70.8–90.2)	0.285	65.0(52.2–85.7)	78.3(59.9–111.6)	0.374	81.2(69.7–134.8)	0.866	0.866
Mixed Meal Tolerance Test									
AUC C-peptide (nmol/L)	473.3(376.9–567.8)	364.2(301.2–483.1)	0.508	421.2(40.9.8–526.9)	337.6(328.9–353.9)	0.139	595.6(460.5–623.8)	570.6(344.6–697.2)	1.000
M_0_ ×10^−8^ (1/min)	− 0.4(− 2.6–0.8)	2.1(− 0.6–3.3)	0.203	−0.5 (− 2.8–1.2)	2.9(0.5–3.2)	0.214	− 2.6(− 5.9–1.2)	0.1(− 3.7–4.3)	0.176
M_1_ × 10^− 8^ (1/min)	6.4(4.5–8.7)	4.6(3.1–5.2)	0.333	5.7(3.6–7.6)	3.3(2.4–4.2)	0.214	7.7(5.5–1.2)	3.8(1.6–8.3)	0.310
Hyperinsulinemic-Euglycemic Clamp Study									
Glucose disposal rate (mg/Kg min)	2.7(2.0–5.5)	2.9(2.7–3.9)	0.508	3.2(2.6–3.5)	3.4(3.1–3.8)	0.173	2.2(1.8–3.0)	2.6(1.8–3.0)	0.866
Insulin sensitivity (mg/(Kgmin)/μU/mL)	4.4(2.5–8.0)	4.0(3.4–5.9)	0.878	5.3(3.6–6.7)	5.6(5.2–6.1)	0.859	3.2(2.3–5.1)	3.4(2.4–4.8)	0.866

All values are expressed as median and interquartile range (1st IQR – 3rd IQR)

*AUC* Area under curve, *M*_*0*_ Basal β-cell function, *M*_*1*_ postprandial β-cell function, *HOMA-IR* Homeostatic model assessment of insulin resistance, and *HOMA-β* Homeostatic model assessment of β cell function

^a^Significant difference from baseline

**Fig. 3:**
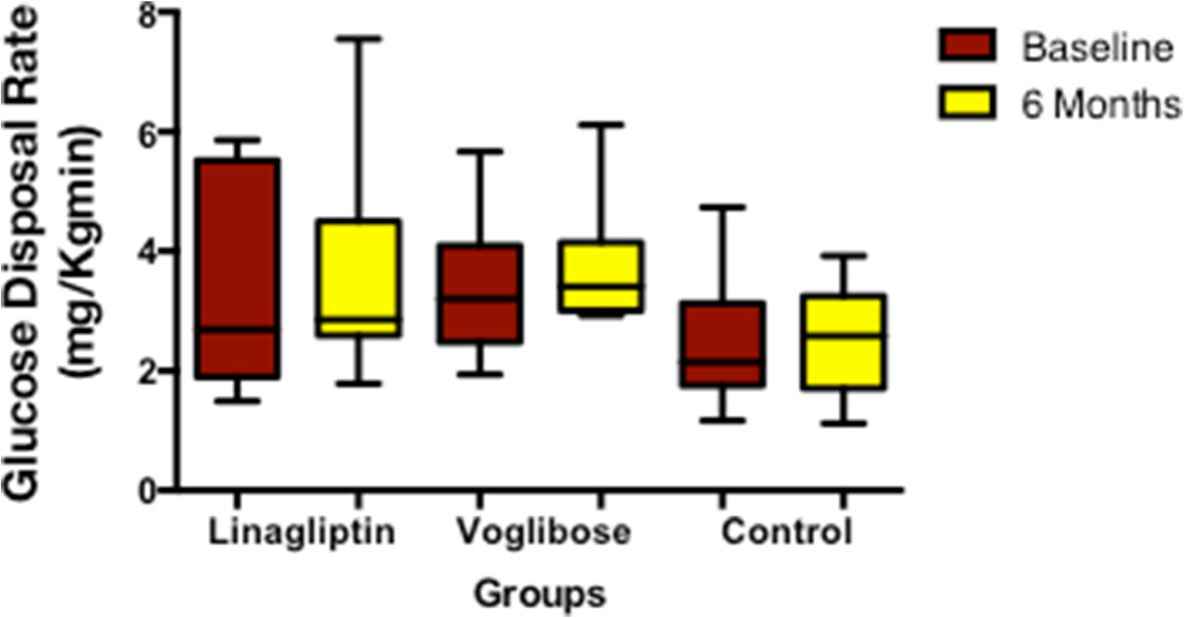
Change in glucose disposal rate among the study groups

**Fig. 4:**
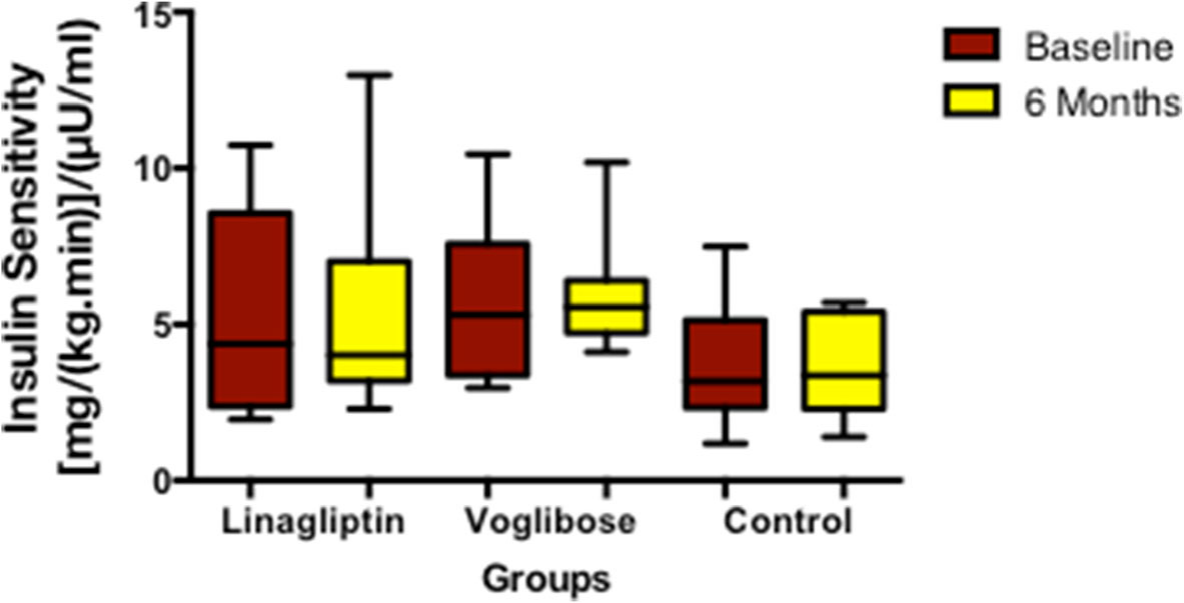
Change in insulin sensitivity among the study groups

Patients treated with linagliptin and voglibose showed a decrease in FPG levels, while FPG increased in the control group. HOMA-IR, HOMA-β, glucose disposal rate, insulin sensitivity, M_0_ and M_1_ response and AUC for C-peptide did not change significantly in any of the study groups after 6 months (Table 2, Figs. 3 and 4).

### Comparison among the groups

Among the groups, there were no significant alterations in clinical parameters at 6 months. HbA1c significantly decreased in the voglibose group as compared to the control group (*p* = 0.038). On mixed meal tolerance test and HEC, there were no significant differences between ΔM_0_, ΔM_1,_ ΔAUC of C-peptide, and Δglucose disposal rate and Δinsulin sensitivity, respectively (Table 3).

**Table 3:** Changes in biochemical parameters among the study groups

Parameters	Linagliptin group (*n* = 10)	Voglibose group (*n* = 9)	Control group (*n* = 7)	*P*-Value Linagliptin V/S Control	*P*-Value Voglibose V/S Control	*P*-Value Voglibose V/S Linagliptin
∆HbA1c (%)	−0.2(− 0.6 to 0.3)	−0.8(− 0.9 to − 0.1)	0.1(− 0.1 to 0.3)	0.422	0.038^a^	0.723
Mixed Meal Tolerance Test						
∆AUC C-pep (nmol/L)	−100.5 (− 174.8 to 75.6)	− 80.3 (− 217.6 to 15.0)	−24.9 (− 153.2184.6)	0.669	0.470	0.780
∆M_0_ × 10^− 8^ (1/min)	1.5(0.2 to 2.7)	3.5(− 1.4 to 3.9)	3.7(0.6 to 6.7)	0.497	0.918	0.475
∆M_1_ ×10^−8^ (1/min)	−2.1(−3.7 to 0.2)	−3.3(−6.4 to 2.1)	−3.8(− 6.1 to 0.6)	0.842	1.000	0.536
Hyperinsulinemic-Euglycemic Clamp Study						
∆Glucose disposal rate (mg/Kg min)	0.5(−0.2 to 0.7)	0.2(0.1 to 0.5)	0.1(−0.4 to 0.4)	0.536	0.470	0.842
∆Insulin sensitivity (mg/(Kgmin)/μU/mL)	0.6(−1.5 to 1.3)	0.2(−0.9 to 0.9)	0.2(−0.8 to 0.7)	0.601	0.905	0.837

All values are expressed as median and interquartile range (1st IQR to 3rd IQR). −Decrease from baseline. ^a^Significant difference within the group

***∆****AUC* Area under curve, ***∆****M*_*0*_ Basal β-cell function, ***∆****M*_*1*_ postprandial β-cell function

## Discussion

Our study demonstrated a modest reduction in HbA1c and insignificant alterations in insulin sensitivity indices in patients with well controlled T2DM who were treated with linagliptin. Our study is a pioneer to demonstrate the effect of linagliptin on insulin sensitivity indices, independent of glucotoxicity, as opposed to the previous studies with DPP 4 inhibitors. In addition, this is the first study in the English literature to evaluate the effect of voglibose on insulin sensitivity using HEC.

In the linagliptin group, HbA1c decreased from 6.8 to 6.6%; however, the difference was not statistically significant. The HbA1c reduction with linagliptin in our study was lower than previously reported, and this may be because of lower HbA1c at baseline (6.8%) [17–19]. It is well known that with any anti-diabetic medication, subjects with higher baseline HbA1c exhibit greater reduction in HbA1c, as compared to those with near-normal glycemia. Moreover, the effect of DPP-4 inhibitors is glucose-dependent; hence, in subjects with near normal HbA1c, their efficacy may be further reduced [20]. There was a significant reduction in HbA1c in the voglibose group at the end of 6 months (from 7.0 to 6.6%). The higher HbA1c reduction observed in our study with voglibose could be due to the dietary habits of the study population, who consume a predominantly carbohydrate-rich diet.

Parameters of insulin resistance assessed by HOMA-IR, glucose disposal rate and insulin sensitivity did not show significant alterations in any of the groups. Improvement in insulin sensitivity with any anti-diabetic agent could be due to its direct insulin sensitizing effect on peripheral tissues or due to reduction in glucotoxicity, or both. Previously, Derosa et al. has shown that both sitagliptin and vildagliptin improved insulin sensitivity in patients with T2DM. [9, 10]. However, the impact of reduction of glucotoxicity on improvement in insulin sensitivity could not be ruled out in these studies, as HbA1c reduced from 8.1 to 6.7% and 8.1 to 6.9%, with the use of sitagliptin and vildagliptin, respectively. In the present study, we recruited patients with well controlled diabetes to ameliorate the effect of glucotoxicity on insulin sensitivity. There was no significant effect of linagliptin on indices of insulin sensitivity as assessed by HEC. Moreover, the significant reduction in HbA1c observed in the voglibose group also did not translate into improvement in insulin sensitivity indicating that further reduction in glucotoxicity (< 7%) may not have a considerable influence on insulin sensitivity.

In the present study, we did not observe any significant alterations in β-cell function indices among participants in any of the groups. This is similar to the results of the study by Retankaran et al., where subjects with HbA1c 7.8 ± 0.8% received intensive insulin therapy for 4–8 weeks and then were randomized to receive either placebo or sitagliptin, with a baseline HbA1c of 6.1 and 6.2%, respectively. Sitagliptin did not demonstrate any improvement in β-cell function at the end of the study [21]. As the effect of DPP-4 inhibitors is glucose-dependent, it is quite possible that these drugs may not exert significant beneficial effect on β-cell function in subjects with well-controlled diabetes. On the contrary, a study by Azuma et al., showed that vildagliptin significantly improved both β-cell function and insulin sensitivity in subjects with a baseline HbA1c 7.1% [22]. This raises the possibility that improvement in insulin sensitivity seen with vildagliptin in those with relatively well-controlled diabetes may be a drug-specific effect, rather than a class effect of DPP 4 inhibitors [22].

The strengths of our study include the presence of a placebo arm, active comparator as voglibose and the use of hyperinsulinemic-euglycemic clamp technique to assess insulin sensitivity. Voglibose was chosen as an active comparator because of its unique mechanism of action, which is insulin-independent, and is not expected to alter insulin sensitivity directly.

There are several limitations in our study. Most importantly, our sample size was small and this prevents us from drawing any meaningful conclusion about the effect of linagliptin on insulin sensitivity. In addition, the duration of study was only 6 months. Since this was a pilot study, we did not calculate the sample size and included 30 patients. Previous studies of DPP 4 inhibitors on insulin sensitivity included larger number of patients and had a follow up of 1 year [9, 10]. However, this was a pilot study to evaluate the effect of linagliptin on insulin sensitivity in patients with well controlled T2DM and more studies involving larger number of patients and longer duration of follow up are needed to conclusively evaluate the effects of linagliptin in patients with well controlled T2DM. We also did not evaluate the presence or severity of nonalcoholic fatty liver disease (NAFLD) in our study subjects, and excluded patients with plasma aminotransferase elevations of more than 3 times upper limit of normal. There is a high prevalence of NAFLD in patients with T2DM [23] .It is well known that NAFLD is closely associated with insulin resistance and subjects with NAFLD have higher insulin resistance as compared to controls [24]. Even though we excluded patients with elevated aminotransferases, NAFLD can be present even in those with normal liver enzymes [25], and this could confound the results of our study. In addition, there was higher number of females in the linagliptin group (5 as compared to 2 in voglibose and placebo groups). Although some studies suggest that women are more insulin resistant as compared to males [26], recent evidence support the fact that women are more insulin sensitive [27, 28]. The higher proportion of females in linagliptin arm could also be a potential confounder in our study. Finally, we used mixed meal test to evaluate pancreatic β-cell function and not the gold standard- hyperglycemic clamp study.

## Conclusion

Linagliptin modestly improves glycemic profile in patients with well controlled T2DM; however, it may not have an effect on insulin sensitivity in these patients.

## Abbreviations

AUC: Area under curve
DPP4: Dipeptidyl peptidase-4
FPG: Fasting plasma glucose
GLP1: Glucagon-like peptide-1
HbA1c: glycated hemoglobin
HEC: Hyperinsulinemic euglycemic clamp
HOMA-IR: Homeostatic model assessment of insulin resistance
HOMA-β: Homeostatic model assessment of β cell function
NAFLD: Nonalcoholic fatty liver disease
OD: Once a day
T2DM: Type 2 diabetes mellitus
TID: Three times a day
